# Association between triglyceride-to-high density lipoprotein cholesterol ratio and three-month outcome in patients with acute ischemic stroke: a second analysis based on a prospective cohort study

**DOI:** 10.1186/s12883-022-02791-2

**Published:** 2022-07-16

**Authors:** Yong Han, Zhiqiang Huang, Jinsong Zhou, Zhibin Wang, Qiming Li, Haofei Hu, Dehong Liu

**Affiliations:** 1 Department of Emergency, Shenzhen Second People’s Hospital, No.3002 Sungang Road, Futian District, Shenzhen, 518035 Guangdong Province China; 2 Department of Laboratory Medicine, Shenzhen Second People’s Hospital, No.3002 Sungang Road, Futian District, Shenzhen, 518035 Guangdong Province China; 3 Department of Nephrology, Shenzhen Second People’s Hospital, No.3002 Sungang Road, Futian District, Shenzhen, 518035 Guangdong Province China

**Keywords:** TG/HDL-c, Acute ischemic stroke, Nonlinear relationship, Generalized additive model, Smooth curve fitting

## Abstract

**Objective:**

Evidence regarding the relationship between serum triglyceride-to-high density lipoprotein cholesterol (TG/HDL-c) ratio and outcomes in acute ischemic stroke (AIS) patients is still mixed. Therefore, the present study was undertaken to explore the link between the TG/HDL-c ratio and unfavorable outcomes in patients with AIS.

**Methods:**

This was a second analysis based on a cohort study. The study population was 1764 patients with AIS collected from January 2010 to December 2016 at a hospital in South Korea. We used a binary logistic regression model to assess the linear association between the TG/HDL-c ratio and unfavorable outcomes for AIS patients. A generalized additive model (GAM) and smooth curve fitting (penalized spline method) was conducted to explore the nonlinear relationship between TG/HDL-c ratio and unfavorable outcomes for AIS patients. Additionally, we compute the inflection point using a recursive algorithm and then build a two-piece binary logistic regression model on both sides of the inflection point. A log-likelihood ratio test was used to determine the most appropriate model describing the association of TG/HDL-c ratio and unfavorable outcomes in patients with AIS.

**Results:**

The incidence rate of unfavorable outcomes was 28.2%, and the median TG/HDL-c ratio was 2.130. After adjusting covariates, the results of the binary logistic regression model suggested that the relationship between the TG/HDL-c ratio and the risk of unfavorable outcomes for AIS patients was not statistically significant. However, there was a nonlinear relationship between them, and the inflection point of the TG/HDL-c ratio was 3.515. On the left side of the inflection point, each 1-unit increase in the TG/HDL-c ratio was associated with a 22.6% lower risk of unfavorable outcomes (OR = 0.774, 95%CI:0.656 to 0.914, *p* = 0.002). On the right side of the inflection point, the effect size (OR) was 1.195 (95%CI:1.004 to1.423, *p* = 0.003).

**Conclusion:**

There is a nonlinear relationship and threshold effect between the TG/HDL-c ratio and 3-month unfavorable outcomes in AIS patients. When the TG/HDL-c ratio is lower than 3.515, the TG/HDL-c ratio is significantly negatively related to the risk of unfavorable outcomes. When the TG/HDL-c ratio is greater than 3.515, the TG/HDL-c ratio was positively associated with the risk of unfavorable outcomes in AIS patients. This provides a reference for optimizing lipidemia intervention and promoting clinical communication in patients with AIS.

**Supplementary Information:**

The online version contains supplementary material available at 10.1186/s12883-022-02791-2.

## Introduction

Stroke constitutes the primary cause of acquired disability in adults. It is the second leading cause of death worldwide, resulting in a substantial economic and social burden on patients and society [1, 2]. Acute ischemic stroke(AIS), resulting from arterial occlusion in the brain, makes up more than 80% of all cases [3]. Accurate prediction of functional outcomes in patients with stroke can enhance clinical care, facilitate education and counseling of patients and families, and simplify recovery and discharge planning [4]. Therefore, identifying prognostic factors in patients with stroke would be of great value in risk stratification, treatment selection, and prognosis assessment*.* The primary known prognostic factors for stroke include age, heart disease, diabetes, stroke etiology, and hypertension [2, 5–8].

Dyslipidemia refers to abnormal lipidemia levels characterized by low levels of high-density lipoprotein cholesterol (HDL-c) and high levels of low-density lipoprotein cholesterol (LDL-c) and triglycerides(TG) [9]. Dyslipidemia is a significant risk factor for cardiovascular and cerebrovascular diseases [10, 11]. Many studies have focused on the association between dyslipidemia and the incidence of ischemic stroke [11, 12]. However, there are few studies on the relationship between dyslipidemia and prognosis in patients with AIS. Recently, an atherogenic dyslipidemia parameter, namely, the triglyceride-to-high density lipoprotein cholesterol (TG/HDL-c) ratio, has been thought to be correlated with cardiovascular events, incident hypertension, and fatty liver [13, 14]. In addition, TG/HDL-c ratio has been shown to hold greater predictive value for cardiovascular disease risk in individuals without symptomatic cardiovascular disease than the isolated lipid parameters used independently [15].

Nevertheless, studies on the effect of TG/HDL-c ratio on clinical outcomes of ischemic stroke are rarely reported, and the results are controversial. One cross-sectional study reported that higher TG/HDL-C levels were associated with reduced 3-month mortality and favorable outcomes in patients with AIS [16]. Another observational study also confirmed that the TG/HDL-C ratio was independently associated with a reduced risk of 3-month mortality in patients with AIS [17]. However, it is known that high levels of TG and low levels of HDL-c are considered risk factors for ischemic stroke [18]. Therefore, we propose a hypothesis that elevated TG/HDL-c ratio may still be a risk factor for unfavorable outcomes in AIS patients. In addition, taking into account the difference in the distribution range of the TG/HDL-c ratio, there may be a nonlinear relationship between TG/HDL-c ratio and unfavorable outcomes in AIS patients. Therefore, we performed an analysis using the published data from a cohort study in South Korea to observe the relationship between TG/HDL-c ratio and unfavorable outcomes in AIS patients.

## Methods

### Study design

This cohort study was conducted using January 2010 and December 2016 records based on a single-center prospective registry system in South Korea [19]. The interesting independent variable in the present work was the TG/HDL-C ratio. The dependent variable was the 3-month outcome in patients with AIS (dichotomous variable: unfavorable outcome, favorable outcome).

### Data source

The data were obtained from this study-Kang MK, Kim TJ, Kim Y, et al.: Geriatric nutritional risk index predicts poor outcomes in patients with acute ischemic stroke-automated undernutrition screen tool [19]. This is an open-access article distributed under the terms of the Creative Commons Attribution License, which permits unrestricted use, distribution, and reproduction in any medium, provided the original author and source are credited [19]. Here, we would like to thank the authors for sharing the data.

### Study population

The original researchers collected South Korean people with AIS admitted within seven days of symptom onset. Data based on a single-center prospective registry system. The original study was conducted with approval from the Institutional Review Board of Seoul National University Hospital. And the Institutional Review Board waived the need for patient consent (IRB No. 1009–062-332). Therefore, the current secondary analysis has not required ethical approval. Furthermore, the original study was conducted in accordance with the declaration of Helsinki; All methods were performed following the relevant guidelines and regulations by including a statement in the declarations section. So did this secondary analysis.

**Fig. 1:**
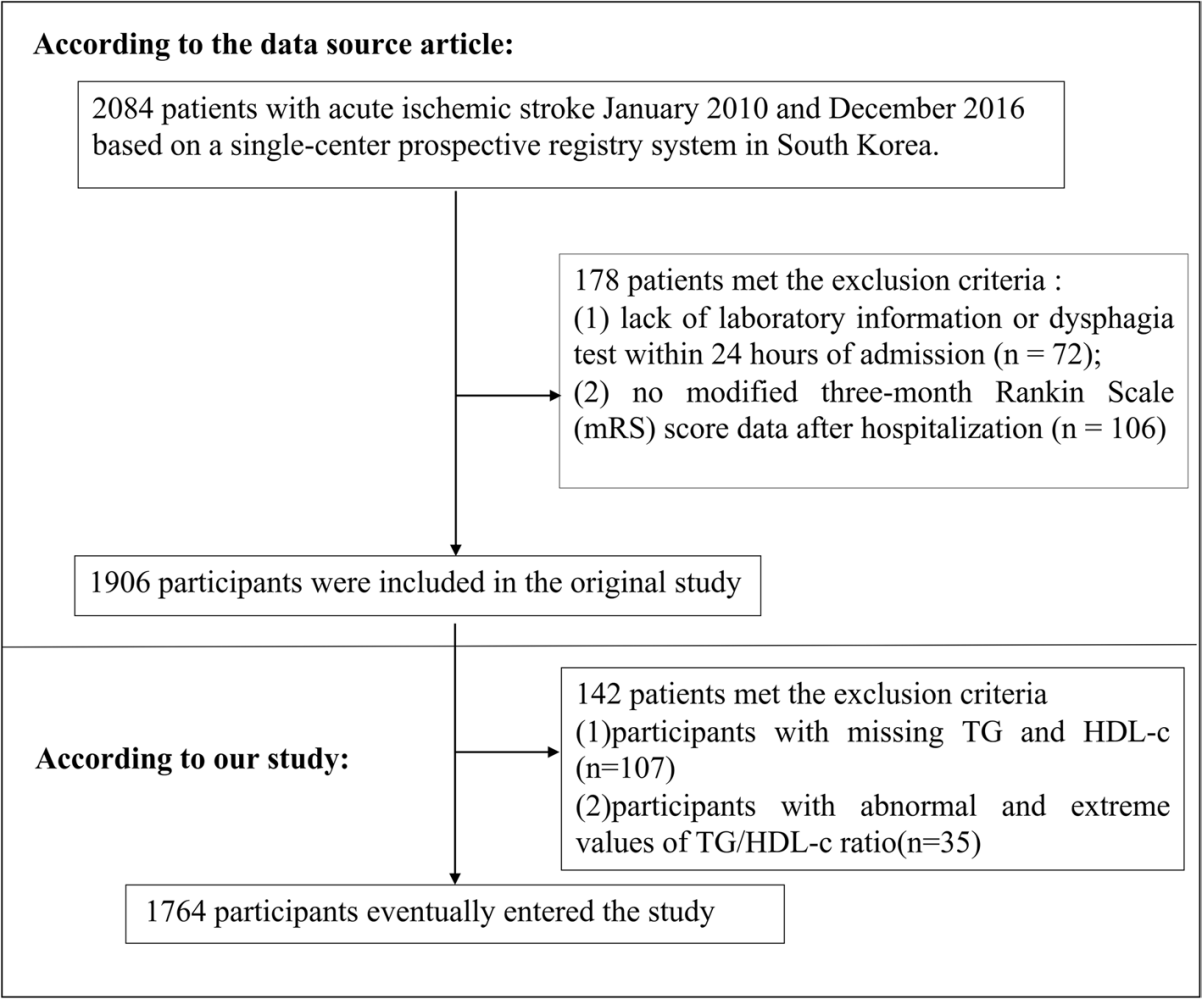
Flowchart of study participants

The original study initially enrolled 2084 South Korean individuals. Afterward, 178 participants were excluded, and 1906 participants were left for data analysis (see flowchart for details in Fig. 1) [19]. In the current study, we further excluded participants with missing TG or HDL-c (*n* = 107) and participants with abnormal and extreme values of TG/HDL-c ratio(greater or less than three standard deviations from the mean) (*n* = 35) [20]. Finally, our study included 1764 participants for secondary analysis. Figure 1 depicts the participant selection process.

### Variables

The TG/HDL-c ratio was recorded as a continuous variable. The detailed process of defining the TG/HDL-c ratio was described as follows: TG/HDL-c ratio = serum triglyceride (mg/dl) divided by serum high-density lipoprotein cholesterol (mg/dl). The TG/HDL-c ratio was converted to categorical variables according to quartiles (Q1: < 1.435, Q2: 1.435–2.125, Q3: 2.125–3.205, and Q4: ≥ 3.205).

### Three-month outcomes in patients with acute ischemic stroke

Three-month outcomes after AIS onset were evaluated using the modified Rankin Scale (mRS) score [21]. Information via outpatient or structured telephone interviews [19]. Participants were divided into two groups with favorable outcomes and unfavorable outcomes. The unfavorable outcome was defined as mRS score ≥ 3; the favorable outcome was defined as mRS score ≤ 2 [21].

### Covariates

Covariates were selected in our study according to the previous literature [16, 17] and our clinical experience. The following variables were used as covariates: (1) continuous variables: hemoglobin* c*oncentration (HGB), hematocrit (HCT), mean corpuscular volume (MCV), platelet (PLT), serum triglyceride (TG), total serum cholesterol (TC), serum high-density lipoprotein cholesterol (HDL-c), serum low-density lipoproteins cholesterol (LDL-c), blood Urea Nitrogen (BUN), serum creatinine (Scr), alanine aminotransferase (ALT), aspartate aminotransferase (AST), serum albumin *(*ALB*)*, fasting blood glucose *(*FBG*)*, body mass index (BMI), fibrinogen(FIB); (2) Categorical variables: age, sex, diabetes mellitus (DM*)*, coronary heart disease (CHD), previous stroke/transient ischemic attack (TIA)*,* hypertension, stroke etiology, national institute of health stroke scale (NIHSS) score, smoking. Laboratory information (test within 24 h of admission) was collected from the electronic medical record [19]. BMI was calculated as weight in kilograms divided by height in meters square (kg/m^2^).

### Missing data processing

In our study, the number of participants with missing data of TC, LDL-c, FIB, and FBG was 1 (0.05%), 9 (0.51%), 16 (0.91%), and 75 (4.25%), respectively. To reduce the deviation caused by missing covariates, which cannot reflect the statistical efficiency of the target sample in the modeling process, the missing data in this study adopts multiple imputations [22, 23]. The imputation model(the type was Linear regression, iterations were 10) included sex, age, HGB, HCT, MCV, PLT, TG, TC, HDL-c, LDL-c, BUN, Scr, ALT, AST, ALB, FBG, BMI, FIB, DM, CHD, previous stroke or TIA, hypertension, stroke etiology, smoking, NIHSS score, 3-month outcomes. Missing data analysis procedures use missing-at-random (MAR) assumptions [23]. It was worth noting that the imputed data were pooled for analysis, and only TC, LDL-C, FIB, and FBG were the variables imputed.

### Statistical analysis

Mean ± standard deviation (SD) (Gaussian distribution) or median (interquartile ranges) (Skewed distribution) were reported for continuous variables, and frequencies and percentages were presented for categorical variables. We used χ2 (categorical variables), the one-way ANOVA test (normal distribution), or the Kruskal–Wallis H test (skewed distribution) to test for differences among different TG/HDL-c ratio groups.

After collinearity screening (Table S1; TC and LDL-c were eliminated), we constructed three distinct models using univariate and multivariate binary logistic regression models to explore the link between TG/HDL-c ratio and unfavorable outcomes in patients with AIS. The models were as follows:(i)a non-adjusted model (no covariates were adjusted); (ii)a minimally-adjusted model (only sociodemographic variables were adjusted, including age and sex); (iii)a fully-adjusted model (adjusted age, sex, HGB, BMI, HCT, AST, BUN, ALB, FBG, FIB, DM, previous stroke or TIA, hypertension, CHD, stroke etiology, smoking, NIHSS score). Effect sizes with 95% confidence intervals(95%CI) were recorded. We adjusted for confounding factors based on clinical experience, literature reports, and the results of univariate analysis.

To test the robustness of our results, we performed a series of sensitivity analyses. We converted the TG/HDL-c ratio into a categorical variable according to the quartile. We calculated the P for the trend to verify the results of the TG/HDL-c ratio as the continuous variable and examine the possibility of the nonlinear relationship between the TG/HDL-c ratio and 3-month unfavorable outcomes (The method used was linear-by-linear association). Since unfavorable outcomes in patients with AIS were significantly associated with DM, abnormal FBG, hypercholesterolemia, and BMI [24–26], we excluded patients with DM, FBG ≥ 6.1 mmol/L, TC ≥ 200 mg/dL, and BMI ≥ 25 kg/m^2^ for sensitivity analysis [27–29]. Additionally, we explored the potential for unmeasured confounding between the TG/HDL-c ratio and unfavorable outcomes by calculating E-values [30].

We further explored the nonlinearity between the TG/HDL-c ratio and unfavorable outcomes using generalized additive models (GAM) and smooth curve fitting (penalized splines). If nonlinearity is detected, we first compute the inflection point using a recursive algorithm (The recursive algorithm begins with an arbitrary initiation followed by filtering/smoothing steps to find the inflection point) and then build a two-piece binary logistic regression model on both sides of the inflection point [31]. A log-likelihood ratio test was used to determine the most appropriate model describing the association of TG/HDL-c ratio and unfavorable outcomes in patients with AIS.

All results were written according to the STROBE statement [32]. Statistical testing was performed using the statistical software packages R (http://www.R-project.org, The R Foundation) and EmpowerStats (http://www.empowerstats.com, X&Y Solutions, Inc, Boston, MA). P values less than 0.05 (two-sided) were considered statistically significant.

## Results

### Characteristics of participants

**Fig. 2:**
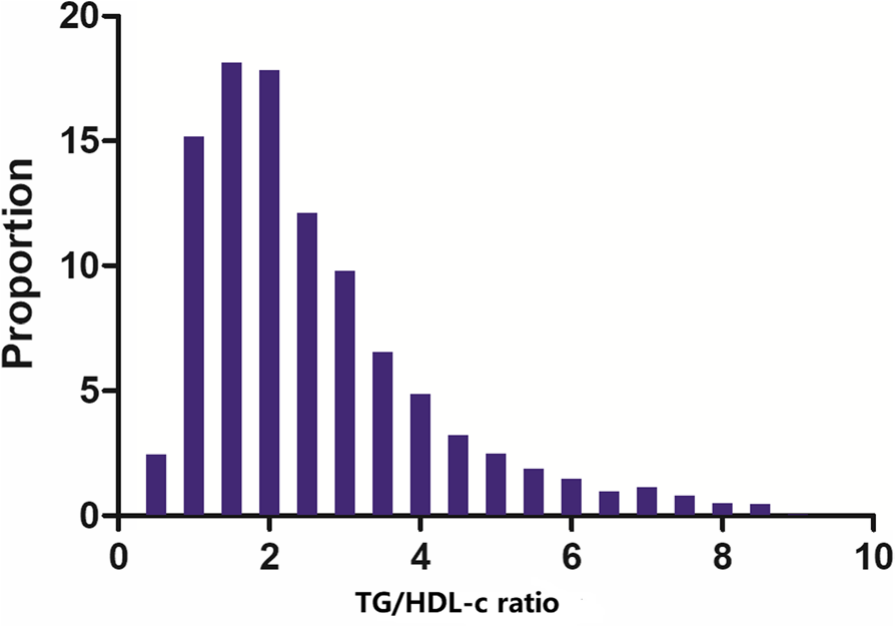
Distribution of TG/HDL-c ratio. It presented a skewed distribution while being in the range from 0.31 to 8.83

Table 1 provided the demographic and clinical characteristics of participants included in the study. 1764 participants were included in the final analysis, of whom 60.83% were male. The number of participants aged < 60, 60 to < 70, 70 to < 80, and ≥ 80 years were 395 (22.39%), 466 (26.42%), 629 (35.66%), and 274 (15.53%), respectively. The number of participants for stroke etiology, including SVO, LAA, CE, other determined, and undetermined, was 343 (19.44%), 574 (32.54%), 448 (25.397%), 148 (8.390%), and 251 (14.229%), respectively. The median (interquartile ranges) of the NHISS score was 3.0 (1.0–7.0). We used TG/HDL-c ratio quartiles to assign participants to subgroups: Q1: < 1.435, Q2: 1.435–2.125, Q3: 2.125–3.205, and Q4: ≥ 3.205, respectively. When we set the TG/HDL-c ratio < 1.435 group as a reference, the values of HGB, PLT, TG, TC, LDL-c, Scr, BMI, and FIB were higher in the ≥ 3.205 group, but lower HDL-c. The fourth quartile (Q4) had a higher proportion of males (33.860%), smoking (48.19%), and DM (36.795%). In addition, the stroke etiologies were more likely to be large artery atherosclerosis or small vessel occlusion in the TG/HDL-c ratio ≥ 3.205 group. Figure 2 showed the distribution of TG/HDL-c ratio levels. It presented a skewed distribution while being in the range from 0.310 to 8.830, with a median of 2.130.

**Table 1:** The baseline characteristics of participants

TG/HDL-ratio (quartile)	Q1(< 1.435)	Q2(1.435 to 2.125)	Q3(2.125 to 3.205)	Q4(≥ 3.205)	*P*-value
participants	441	440	441	442	
HGB(g/dL)	13.24 ± 1.82	13.56 ± 1.85	13.60 ± 1.93	13.64 ± 2.20	0.010
HCT(%)	39.57 ± 5.06	40.33 ± 5.19	40.44 ± 5.36	40.38 ± 6.17	0.059
MCV*(*fl)	93.29 ± 5.65	93.40 ± 5.28	92.92 ± 4.58	92.36 ± 5.25	0.013
PLT(10^9/L)	218.56 ± 64.95	220.64 ± 69.24	222.61 ± 69.06	236.74 ± 77.58	< 0.001
TC(mg/dl)	176.74 ± 39.28	180.40 ± 43.62	177.46 ± 42.04	184.80 ± 46.73	0.022
TG(mg/dl)	63.77 ± 15.23	88.20 ± 16.80	109.91 ± 23.56	167.31 ± 50.97	< 0.001
HDL-c(mg/dl)	60.28 ± 12.47	49.71 ± 9.85	42.16 ± 8.63	36.04 ± 8.03	< 0.001
LDL-c(mg/dl)	100.10 ± 33.30	110.58 ± 38.77	111.24 ± 36.08	113.33 ± 39.41	< 0.001
BUN(mg/dl)	15.00 (12.00–20.00)	15.00 (12.00–20.00)	16.00 (13.00–20.00)	15.00 (12.00–20.00)	0.655
Scr(mg/dl)	0.85 (0.70–1.00)	0.88 (0.73–1.08)	0.90 (0.76–1.05)	0.95 (0.76–1.16)	< 0.001
ALT(U/L)	23.00 (19.00–29.00)	23.00 (18.00–29.00)	23.00 (19.00–29.00)	23.00 (18.00–29.75)	0.467
AST (U/L)	17.00 (13.00–24.00)	17.00 (12.75–25.00)	19.00 (14.00–26.00)	19.00 (14.00–29.00)	0.090
ALB (g/dL)	4.04 ± 0.36	4.04 ± 0.41	4.03 ± 0.43	4.00 ± 0.45	0.448
FBG*(*mmol/L*)*	5.28 (4.67–6.28)	5.44 (4.66–6.62)	5.44 (4.83–6.44)	5.44 (4.83–6.72)	0.059
FIB (m*g*/L)	321.96 ± 69.92	326.11 ± 78.89	333.42 ± 83.83	350.25 ± 94.70	< 0.001
BMI (kg/m^2^)	22.46 ± 2.99	23.22 ± 3.19	23.85 ± 3.20	24.47 ± 3.21	< 0.001
Sex					< 0.001
Male	224 (50.79%)	266 (60.45%)	291 (65.99%)	292 (66.06%)	
Female	217 (49.21%)	174 (39.55%)	150 (34.01%)	150 (33.94%)	
Age(years)					0.006
< 60	91 (20.63%)	83 (18.86%)	103 (23.36%)	118 (26.70%)	
60 to < 70	107 (24.26%)	120 (27.27%)	124 (28.12%)	115 (26.02%)	
70 to < 80	153 (34.69%)	160 (36.36%)	162 (36.73%)	154 (34.84%)	
≥ 80	90 (20.41%)	77 (17.50%)	52 (11.79%)	55 (12.44%)	
Previous stroke/TIA	91 (20.63%)	97 (22.05%)	89 (20.18%)	92 (20.81%)	0.917
Hypertension	261 (59.18%)	282 (64.09%)	284 (64.40%)	289 (65.38%)	0.222
DM	106 (24.04%)	135 (30.68%)	147 (33.33%)	163 (36.88%)	< 0.001
Smoking	120 (27.21%)	166 (37.73%)	202 (45.80%)	213 (48.19%)	< 0.001
CHD	39 (8.84%)	55 (12.50%)	55 (12.47%)	54 (12.22%)	0.249
Stroke etiology					< 0.001
SVO	80 (18.14%)	90 (20.45%)	86 (19.50%)	87 (19.68%)	
LAA	124 (28.12%)	136 (30.91%)	145 (32.88%)	169 (38.24%)	
CE	142 (32.20%)	119 (27.05%)	103 (23.36%)	84 (19.00%)	
Other determined	23 (5.22%)	39 (8.86%)	48 (10.88%)	38 (8.60%)	
Undetermined	72 (16.33%)	56 (12.73%)	59 (13.38%)	64 (14.48%)	
NIHSS score					0.273
< 6	285 (64.63%)	306 (69.55%)	303 (68.71%)	315 (71.27%)	
6 to 13	93 (21.09%)	82 (18.64%)	93 (21.09%)	84 (19.00%)	
≥ 14	63 (14.29%)	52 (11.82%)	45 (10.20%)	43 (9.73%)	

Values are mean ± standard deviation or median (quartile) or number (%)

*HGB* Hemoglobin concentration, *HCT* Hematocrit, *MCV* Mean corpuscular volume, *PLT* Platelet, *TG* Triglyceride, *TC* Total cholesterol, *HDL-c* High-density lipoprotein cholesterol, *LDL-c* Low-density lipoproteins cholesterol, *BUN* Blood urea nitrogen, *Scr* Serum creatinine*, ALT* Alanine aminotransferase, *AST* Aspartate aminotransferase, *ALB* Serum albumin*, FBG* Fasting blood glucose, *FIB* Fibrinogen*, BMI* body mass index, *DM* Diabetes mellitus, *CHD* Coronary heart disease, *TIA* Transient ischemia attack. *LAA* Large artery atherosclerosis, *SVO* Small vessel occlusion, *CE* Cardio embolism, *NIHSS* National institute of health stroke scale, *TG/HDL* Ratio, *triglyceride* -to- high density lipoprotein cholesterol ratio

### The incidence rate of unfavorable outcomes 3-month after acute ischemic stroke

**Fig. 3:**
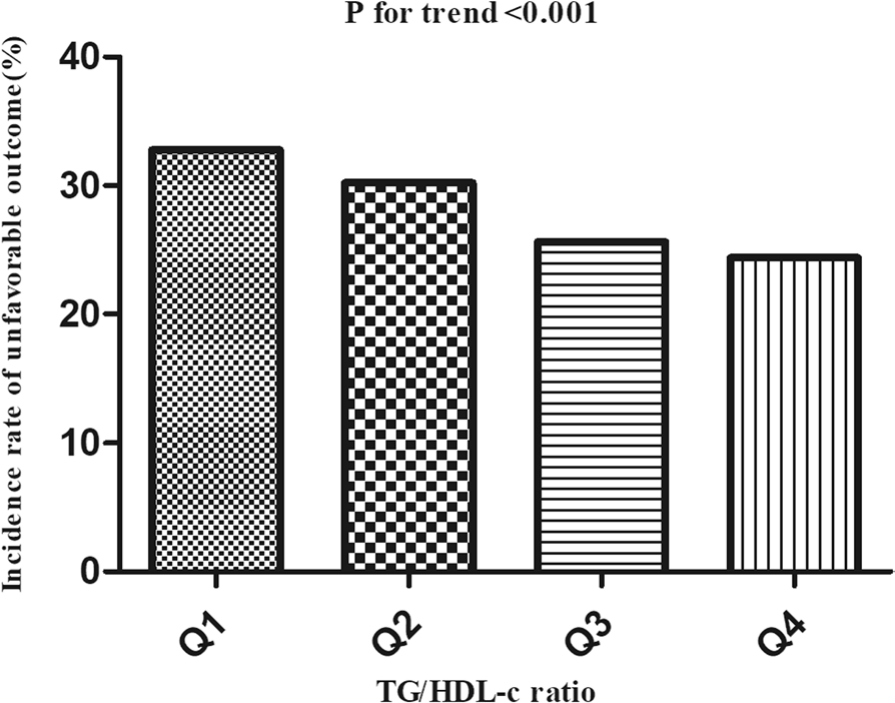
Incidence of unfavorable outcomes according to the quartiles of TG/HDL-c ratio

Table 2 revealed that 498 participants had unfavorable outcomes in total. The total incidence rate of unfavorable outcomes was 28.2% (26.1%-30.3%). Specifically, the incidence rate of unfavorable outcomes for the TG/HDL-c ratio quartiles was Q1: 32.8% (28.4%-37.2%), Q2: 30.2% (26.0%-34.5%), Q3: 25.6% (21.5%-29.6%) and Q4: 24.4% (20.4%-28.4%), respectively. Compared with the lowest TG/HDL-c ratio group (Q1), participants with a high TG/HDL-c ratio (Q4) had a lower incidence rate of unfavorable outcomes (*p* < 0.001 for trend) (Fig. 3).

**Table 2:** Incidence rate of unfavorable outcome 3-month after stroke

TG/HDL ratio	Participants(n)	unfavorable outcome events (mRS score ≥ 3)	Incidence of unfavorable outcome (95% CI)(%)
Total	1764	498	28.2(26.1–30.3)
Q1	436	143	32.8(28.4–37.2)
Q2	443	134	30.2(26.0–34.5)
Q3	442	113	25.6(21.5–29.6)
Q4	443	108	24.4 (20.4–28.4)
P for trend			< 0.001

*TG/HDL-c* *Ratio* triglyceride -to- high-density lipoprotein ratio cholesterol, *mRS* modified Rankin scale;

### The results of univariate analyses using a binary logistic regression model

The univariate analyses showed that the risk of unfavorable outcomes in AIS patients had nothing to do with MCV (odds ratio OR = 0.985, 95%CI: 0.966–1.005), PLT (OR = 1.000, 95%CI:0.998–1.001), Scr (OR = 0.992, 95%CI:0.894–1.101), ALT (OR = 1.007, 95%CI:0.999–1.014), CHD (OR = 0.974, 95%CI:0.702–1.350), HDL-c (OR = 0.997, 95%cCI:0.989–1.005), and LDL-c (OR = 0.998,95%CI:0.995–1.001) (all *P* > 0.05), but was positively related to BUN (OR = 1.014, 95%CI:1.002–1.027), FBG (OR = 1.174, 95%CI:1.118–1.233), FIB (OR = 1.003, 95%CI:1.002–1.004) (all *P* < 0.05). In addition, participants with age ≥ 80 years old (OR = 4.379, 95%CI:3.085–6.218), hypertension (OR = 1.376, 95%CI:1.103–1.716), DM (OR = 1.510, 95%CI:1.213–1.878), previous stroke/TIA (OR = 1.764, 95%CI:1.38–2.248), NHISS score ≥ 14 (OR = 17.026, 95%CI:11.994–24.170), and other determined of stroke etiology had a higher risk of unfavorable outcomes (all *P* < 0.05). However, HGB (OR = 0.823, 95%CI:0.780–0.868), HCT(OR = 0.934, 95%CI:0.916–0.952), AST (OR = 0.990, 95%CI:0.982–0.997), ALB (OR = 0.277, 95%CI:0.213–0.361), BMI (OR = 0.920, 95%CI:0.890–0.951), TG/HDL-c ratio (OR = 0.932, 95%CI:0.869–0.999), TC (OR = 0.996, 95%CI:0.994–0.999), and TG (OR = 0.995, 95%CI:0.993–0.998) was negatively associated with the risk of unfavorable outcomes (see Table 3 for details).

**Table 3:** Influencing factors of unfavorable outcomes in acute ischemic stroke using univariate regression analysis

Variable	Characteristics	OR 95%CI	P
HGB(g/dl), mean ± sd	13.510 ± 1.961	0.823 (0.780, 0.868)	< 0.001
HCT(%),mean ± sd	40.177 ± 5.468	0.934 (0.916, 0.952)	< 0.001
MCV(fl), mean ± sd	92.992 ± 5.215	0.985 (0.966, 1.005)	0.146
PLT(10^9/L), mean ± sd	224.648 ± 70.666	1.000 (0.998, 1.001)	0.736
TC(mg/dl), mean ± sd	179.853 ± 43.085	0.996 (0.994, 0.999)	0.002
TG(mg/dl), mean ± sd	107.344 ± 48.850	0.995 (0.993, 0.998)	< 0.001
HDL-c(mg/dl), mean ± sd	47.040 ± 13.401	0.997 (0.989, 1.005)	0.467
LDL-c(mg/dl), mean ± sd	108.813 ± 37.292	0.998 (0.995, 1.001)	0.112
BUN(mg/dl),median (quartile)	16.000 (12.000–20.000)	1.014 (1.002, 1.027)	0.020
Scr(mg/dl),median (quartile)	0.890 (0.740–1.070)	0.992 (0.894, 1.101)	0.875
ALT(U/L),median (quartile)	23.000 (19.000–29.000)	1.007 (0.999, 1.014)	0.078
AST(U/L),median (quartile)	18.000 (13.000–26.000)	0.990 (0.982, 0.997)	0.008
ALB (g/dL), mean ± sd	4.029 ± 0.413	0.277 (0.213, 0.361)	< 0.001
FBG(mmol/L), mean ± sd	5.920 ± 2.081	1.174 (1.118, 1.233)	< 0.001
TG/HDL-c ratio,median (quartile)	2.128 (1.440–3.208)	0.932 (0.869, 0.999)	0.047
FIB (m*g*/L), mean ± sd	332.946 ± 82.970	1.003 (1.002, 1.004)	< 0.001
BMI(kg/m^2^), mean ± sd	23.499 ± 3.233	0.920 (0.890, 0.951)	< 0.001
Sex, n(%)			
Male	1073 (60.828%)	Ref	
Female	691 (39.172%)	1.747 (1.415, 2.156)	< 0.001
Age(years), n(%)			
< 60	395 (22.39%)	Ref	
60 to < 70	466 (26.42%)	1.301 (0.927, 1.826)	0.128
70 to < 80	629 (35.66%)	2.586 (1.931, 3.462)	< 0.001
≥ 80	203 (11.51%	4.379 (3.085, 6.218)	< 0.001
Hypertension, n(%)			
NO	648 (36.735%)	Ref	
Yes	1116 (63.265%)	1.376(1.103, 1.716)	0.005
DM, n(%)			
No	1213 (68.764%)	Ref	
Yes	551 (31.236%)	1.510 (1.213, 1.878)	< 0.001
Previous stroke/TIA, n(%)			
No	1395 (79.082%)	Ref	
Yes	369 (20.918%)	1.764 (1.384, 2.248)	< 0.001
NIHSS score, n(%)			
< 6	1209 (68.537%)	Ref	
6 to 13	352 (19.955%)	6.774 (5.193, 8.837)	< 0.001
≥ 14	203 (11.508%)	17.026 (11.994, 24.170)	< 0.001
CHD, n(%)			
No	1561 (88.492%)	Ref	
Yes	203 (11.508%)	0.974 (0.702, 1.350)	0.873
Stroke etiology, n(%)			
SVO	343 (19.444%)	Ref	
LAA	574 (32.540%)	1.659 (1.193, 2.306)	0.003
CE	448 (25.397%)	2.351 (1.681, 3.289)	< 0.001
Other determined	148 (8.390%)	3.204 (2.093, 4.905)	< 0.001
Undetermined	251 (14.229%)	1.366 (0.916, 2.037)	0.126
Smoking, n(%)			
No	1063 (60.261%)	Ref	
Yes	701 (39.739%)	0.604 (0.484, 0.752)	< 0.001

*SD* Standard deviation, *n* number

*HGB* Hemoglobin concentration, *HCT* Hematocrit, *MCV* Mean corpuscular volume, *PLT* Platelet*, TG* Triglyceride, *TC* Total cholesterol, *HDL-c* High-density lipoprotein cholesterol, *LDL-c* Low-density lipoproteins cholesterol, *BUN* Blood urea nitrogen, *Scr* Serum creatinine*, ALT* Alanine aminotransferase, *AST* Aspartate aminotransferase, *ALB* Serum albumin*, FBG* Fasting blood glucose, *FIB* Fibrinogen, *BMI* Body mass index, *DM* Diabetes mellitus*, CHD* Coronary heart disease, *TIA* Transient ischemia attack, *LAA* Large artery atherosclerosis, *SVO* Small vessel occlusion, *CE* Cardio embolism, *NIHSS* National institute of health stroke scale, *TG/HDL ratio* Triglyceride *-*to- high-density lipoprotein cholesterol ratio

### The results of multivariate analyses using the binary logistic regression model

We constructed three models using the binary logistic regression model to investigate the association between the TG/HDL-c ratio and the risk of unfavorable outcomes in patients with AIS. In the crude model, a 1-unit increase in the TG/HDL-c ratio was associated with a 6.8% decrease in the risk of unfavorable outcomes (OR = 0.932, 95%CI 0.869–1.000, *p* = 0.049). The results were statistically significant. However, in the minimally adjusted and fully adjusted models, the multivariate regression analysis results were not statistically significant (all *p* > 0.05) (Table 4).

**Table 4:** Relationship between TG/HDL-c ratio and unfavorable outcome 3-month after stroke in different models

Variable	Crude model (OR,95%CI)	P	Model I(OR,95%CI)	P	Model II(OR,95%CI)	P
TG/HDL-c ratio	0.932 (0.869, 0.999)	0.047	0.968 (0.901, 1.041)	0.383	0.950 (0.869, 1.039)	0.260
TG/HDL-c ratio (quartile)						
Q1(< 1.435)	Ref		Ref		Ref	
Q2(1.435 to 2.125)	0.913 (0.687, 1.213)	0.529	0.969 (0.722, 1.300)	0.833	0.933 (0.658, 1.324)	0.698
Q3(2.125 to 3.205)	0.669 (0.498, 0.898)	0.007	0.772 (0.570, 1.047)	0.097	0.655 (0.453, 0.946)	0.024
Q4(≥ 3.205)	0.667 (0.497, 0.895)	0.007	0.770 (0.568, 1.045)	0.094	0.649 (0.444, 0.949)	0.026
P for trend	0.001		0.041		0.007	

Crude mode1: we did not adjust other covariates

Model I: we adjusted age, sex

Model II: we adjusted age, sex, HGB, BMI, HCT, AST, BUN, ALB, FBG, FIB, DM, previous stroke or TIA, hypertension, CHD, stroke etiology, smoking, and NIHSS score

### Sensitivity analysis

We also performed a series of sensitivity analyses. We first converted TG/HDL-c ratio from a continuous variable to a categorical variable (according to quartile) and then put the categorical-transformed TG/HDL-c ratio back into the model. The results of the multivariate-adjusted model showed that with reference to the first quartile (Q1) of the TG/HDL-c ratio, the OR of the third quartile (Q3) was 0.655(95%CI:0.453 to 0.946), while the fourth quartile (Q4) with an OR of 0.649 (95%CI:0.444 to 0.949) (Table 4 Model II). The similar OR for Q3 and Q4 suggested the possibility of a nonlinear relationship between the TG/HDL-c ratio and unfavorable outcomes for AIS patients. In addition, we generated an E-value to assess the sensitivity to unmeasured confounding. The E-value(1.26) was greater than the relative risk (1.15) of unmeasured confounders and TG/HDL-c ratio, suggesting unmeasured or unknown confounders had little effect on the relationship between TG/HDL-c ratio and unfavorable outcome risk of AIS patients.

Furthermore, we excluded participants with TC ≥ 200 mg/dl, DM, FBG ≥ 6.1 mmol/L, and BMI ≥ 25 kg/m^2^ in other sensitivity analyses. The results showed that the linear relationship between the TG/HDL-c ratio and the risk of unfavorable outcomes was still not statistically significant after adjusting for confounders (all *p* > 0.05) (Table 5). Therefore, the linear relationship between the TG/HDL-c ratio and the risk of unfavorable outcomes did not hold either in the overall population or in specific subgroups. We adjusted for all factors in sensitivity analyses, including age, HGB, sex, BMI, HCT, AST, BUN, ALB, FBG, FIB, DM, previous stroke or TIA, hypertension, CHD, stroke etiology, smoking, and NIHSS score. It was worth noting that the adjusted covariates that did not include DM in the model I (participants without DM).

**Table 5:** Relationship between TG/HDL-c ratio and unfavorable outcome in different sensitivity analyses

TG/HDL-c ratio	OR 95%CI	*P*-value
Model I	0.902 (0.799, 1.017)	0.093
Model II	0.940 (0.843, 1.048)	0.268
Model III	0.990 (0.887, 1.104)	0.856
Model IV	0.922 (0.829, 1.026)	0.136

Model I was sensitivity analysis in participants without DM. We adjusted age, sex, HGB, BMI, HCT, AST, BUN, ALB, FBG, FIB, previous stroke or TIA, hypertension, CHD stroke etiology, smoking, and NIHSS score

Model II was sensitivity analysis in participants without BMI ≥ 25 kg/m^2^. We adjusted age, sex, HGB, BMI, HCT, AST, BUN, ALB, FBG, FIB, DM, previous stroke or TIA, hypertension, CHD, stroke etiology, smoking, and NIHSS score

Model III was sensitivity analysis in participants without TC ≥ 200 mg/L. We adjusted age, sex, HGB, BMI, HCT, AST, BUN, ALB, FBG, FIB, DM, previous stroke or TIA, hypertension, CHD stroke etiology, smoking, and NIHSS score

Model IV was sensitivity analysis in participants without FBG ≥ 6.1 mmol/L. We adjusted age, HGB, sex, BMI, HCT, AST, BUN, ALB, FBG, FIB, DM, previous stroke or TIA, hypertension, CHD, stroke etiology, smoking, and NIHSS score

*OR* Odds ratios, *CI* Confidence, *Ref* Reference, *TG/HDL ratio* Triglyceride -to- high-density lipoprotein cholesterol ratio

### The nonlinearity addressed by the generalized additive model

**Fig. 4:**
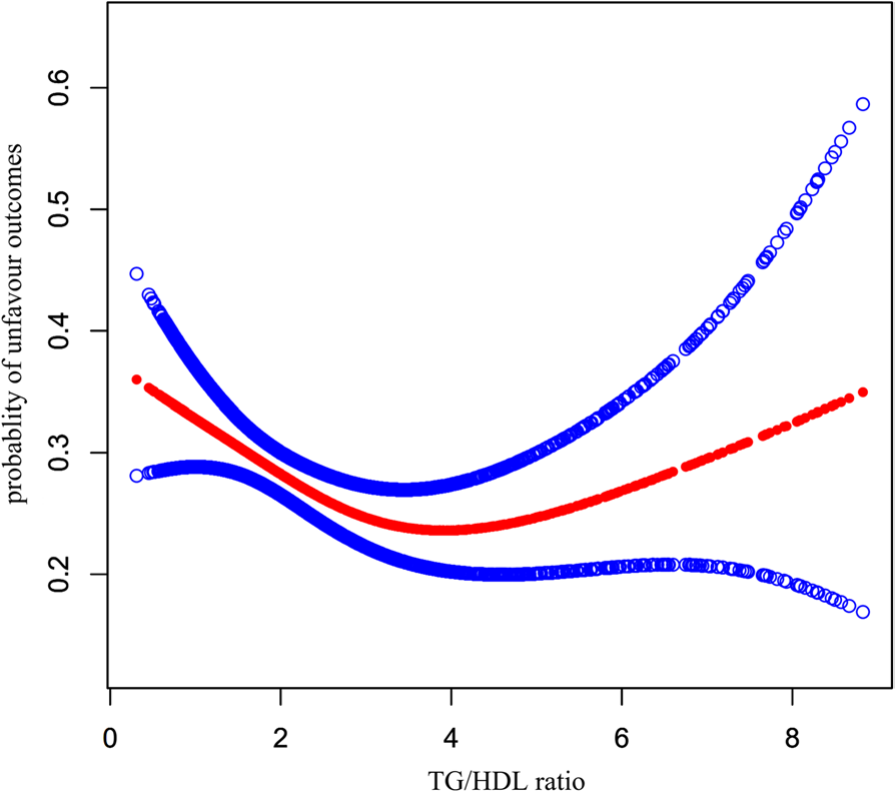
The nonlinear relationship between TG/HDL-c ratio and the risk of unfavorable outcomes. The nonlinear relationship between TG/HDL-c ratio and the risk of unfavorable outcomes. A nonlinear relationship was detected after adjusting for age, gender, HGB, BMI, HCT, AST, BUN, ALB, FBG, FIB, DM, Previous stroke or TIA, hypertension, CHD, stroke etiology, smoking, and NIHSS score

The results of multivariate models based on logistic regression were not statistically significant and may be affected by nonlinear relationships. Additionally, multivariate-adjusted models with TG/HDL-c ratio quartiles as categorical variables suggested similar OR for the third quartile (Q3) and fourth quartile(Q4). These results indicated that there might be a nonlinear relationship between TG/HDL-c ratio and unfavorable outcomes for AIS patients. Through the GAM and smooth curve fitting(adjusted age, sex, HGB, BMI, HCT, AST, BUN, ALB, FBG, FIB, DM, previous stroke or TIA, hypertension, CHD, stroke etiology, smoking, NIHSS score), we observed an approximate “U”-shaped relationship between the TG/HDL-c ratio and unfavorable outcomes risk in AIS patients (Fig. 4). For that reason, we fitted data to a piecewise binary logistic regression model to fit two different slopes. We also fitted data by standard binary logistic regression model based on the sensitivity analysis and selected the best fit model through the log-likelihood ratio test. In our study, the P for the log-likelihood ratio test was less than 0.05. By recursive algorithm, we first obtained the inflection point was 3.515 and then calculated the effect sizes and confidence interval on the left and right of the inflection point by the two-piecewise binary logistic regression model. On the left side of the inflection point, each 1-unit increase in the TG/HDL-c ratio was associated with a 22.6% lower risk of unfavorable outcomes (OR = 0.774, 95%CI:0.656 to 0.914, *p* = 0.002). On the right side of the inflection point, the effect size (OR) was 1.195 (95%CI:1.004 to1.423, *p* = 0.003) (Table 6).

**Table 6:** The result of two-piecewise linear regression model

Unfavorable outcome:	OR(95%CI,)	P
Fitting model by standard linear regression	0.950 (0.869, 1.039)	0.2595
Fitting model by two-piecewise linear regression		
Inflection point of TG/HDL-c ratio	3.515	
≤ 3.515	0.774 (0.656, 0.914)	0.002
> 3.515	1.195 (1.004, 1.423)	0.045
P for log-likelihood ratio test	0.004	

We adjusted age, sex, HGB, BMI, HCT, AST, BUN, ALB, FBG, FIB, DM, previous stroke or TIA, hypertension, CHD, stroke etiology, smoking, and NIHSS score

*OR* Odds ratios, *CI* Confidence, *Ref* Reference;

## Discussion

The current study was designed to examine the link between TG/HDL-c ratio and 3-month outcomes in patients with AIS. We found a nonlinear rather than linear relationship between them. In addition, we found a threshold effect with an inflection point of 3.515 for the TG/HDL-c ratio.

Through a literature search, we found a few pieces of literature currently investigating the relationship between TG/HDL-c ratio and outcomes in patients with AIS. A cross-sectional study of 1006 patients with AIS showed that compared with participants with low TG/HDL-c (< 0.87), participants with high TG/HDL-C (≥ 0.87)had a 66% reduction in 3-month mortality (OR = 0.34, 95%CI:0.22–0.53, *P* < 0.001). The TG/HDL-C ratio was positively associated with favorable outcomes (OR = 1.01, 95%CI 0.22–0.53, *P* < 0.001) [16]. In another cohort study of 1459 patients with AIS, multivariate logistic regression analysis showed that TG/HDL-c ratio was independently associated with reduced mortality (HR = 0.39; 95%CI 0.24 to 0.62; *P* < 0.001) [17]. However, Our findings showed that the relationship between TG/HDL-C ratio and unfavorable outcome based on logistic regression analysis was not statistically significant. The following possible explanations caused these inconsistent findings: First, there may be differences in the distribution of TG/HDL-c ratio among these studies. Our study has a wider distribution of TG/HDL-c ratio. Second, the sample sizes of these studies vary. Third, the outcome variables were different. Previous studies have focused on favorable outcomes or 3-months mortality. And our dependent variable was 3-months unfavorable outcomes in AIS patients (mRS score ≥ 3). Meanwhile, the sensitivity analysis found that in participants with TC < 200 mg/dl, FBG < 6.1 mmol/L, BMI < 25 kg/m^2^, or without DM, the linear relationship between TG/HDL-c ratio and unfavorable outcomes in patients with AIS was still not established.

However, after categorizing the TG/HDL-c ratio variables according to quartiles, the results of the multivariate-adjusted model showed that taking the first quartile (Q1) as a reference, the OR values of the second (Q2), third (Q3), and fourth (Q4) quartile was 0.933, 0.655 and 0.649, respectively. The overall downward trend from Q1 to Q4, but similar results in Q3 and Q4. That is to say, the downward trend of the OR value from Q1 to Q3 is obviously down, and it is terminated in the range of Q3 to Q4. This suggested that there may be a nonlinear relationship between the TG/HDL-c ratio and unfavorable outcomes in AIS patients.

Furthermore, previous studies have found an inverse linear relationship between the TG/HDL-c ratio and the risk of unfavorable outcomes [16, 17]. But another prospective observational study showed that elevated TG levels (≥ 150 mg/dL) and low HDL-c levels (< 40 mg/dL) were associated with 1-year poor outcomes (cardiovascular events, intracranial arteries, and stroke recurrence) in patients with ischemic stroke [33]. These studies suggest that the relationship between TG/HDL-c ratio and unfavorable may be significantly different between participants with lower TG/HDL-c ratio and those with higher TG/HDL-c ratio. In other words, there may be a nonlinear relationship between the TG/HDL-c ratio and the risk of unfavorable outcomes in AIS patients. In the present study, we tested our hypothesis using logistic regression with cubic spline functions and smooth curve fitting (the cubic spline smoothing). Ultimately, we observed that the association between the TG/HDL-c ratio and unfavorable outcome risk was nonlinear. In addition, we got the inflection point was 3.515 and then calculated the OR and CI on the left and right of the inflection point by the two-piecewise logistic regression model. On the left side of the inflection point (≤ 3.515), the OR (95%CI) was 0.774 (0.656–0.914) (Table S2). We further analyzed the relationship between the TG/HDL-c ratio and the risk of unfavorable outcomes in the population with TG/HDL-c ratio > 3.515. The results suggested that on the right side of the inflection point, the OR (95%CI) was 1.195 (1.004, 1.423). That is, when the TG/HDL-c ratio was > 3.515, as the TG/HDL-c ratio increased, the risk of unfavorable outcomes was no longer correspondingly reduced but increased accordingly. Therefore, the nonlinear relationship explored based on a population with a wider distribution of TG/HDL-c ratio may be closer to the actual relationship between TG/HDL-c ratio and unfavorable outcomes in AIS patients. Besides, a nonlinear relationship is a type of relationship between two variables in which change in one entity does not correspond with constant change in the other variable. This might mean the relationship between the two variables seems unpredictable or virtually absent. However, nonlinear entities can be related to each other in ways that are fairly predictable but simply more complex than in a linear relationship. Because of the complexity of the relationship between TG/HDL-c ratio and unfavorable outcomes in AIS patients. Therefore, the nonlinear relationship may also be closer to the relationship between TG/HDL-c ratio and unfavorable outcomes risk. This provides a reference for the clinical optimization of blood lipid intervention in patients with AIS. When the patients’ TG/HDL-c ratio is less than 3.515, we do not need to intervene to reduce TG or increase HDL-c levels actively. When the patient’s TG/HDL-c ratio is greater than 3.515, aggressive intervention is required for high TG or low HDL-c levels.

In addition, the inflection point (3.515) for the TG/HDL-c ratio appears to be high, with clinically fewer patients having a TG/HDL-c ratio above the inflection point. A prospective observational study showed that elevated TG levels (≥ 150 mg/dL) and low HDL-c (< 40 mg/dL) was associated with 1-year poor outcomes in patients with AIS [33]. It suggested that when TG/HDL-c ratio > 3.75(150/40), TG/HDL-c ratio was positively associated with unfavorable outcomes. In addition, 360 (20.41%) participants in this study had a TG/HDL-c ratio > 3.515. In this population with a TG/HDL-c ratio > 3.515, the TG/HDL-c ratio was also positively associated with unfavorable outcomes in stroke patients. Therefore, we tend to think that a higher inflection point for TG/HDL-c may be appropriate.

However, the reasons for the nonlinear relationship between the TG/HDL-c ratio and the risk of unfavorable outcomes in patients with AIS are unclear. Firstly, low serum TG is an indicator of malnutrition, which may affect neurological repair. Besides, acute ischemic stroke is a state of metabolic stress with high-energy requirements. TG stores excess calories and provides energy in necessary metabolic stress conditions. Therefore, lower levels of TG may also be detrimental to early nerve repair in AIS patients [34, 35]. Secondly, a study showed a high HDL-c concentration was associated with an increased risk of unfavorable outcomes [16]. Therefore, on the left side of the inflection point, the TG/HDL-c ratio was inversely related to the risk of unfavorable outcomes, which may be mainly due to too low TG levels or high HDL-c levels. On the right side of the inflection point, as in most other studies, higher TG or lower HDL-c levels became risk factors for unfavorable outcomes in patients with AIS [36]. Elevated TG/HDL-C ratio levels increase the risk of insulin resistance(IR), DM, cardiovascular disease, and hypertension, which are associated with unfavorable outcomes in patients with AIS [13, 14, 37].

Our study has some strengths, and we listed them as follows. First, the independent variables in our study used both the quartile of TG/HDL-c ratio as a categorical variable and a continuous variable of TG/HDL-c ratio to explore its relationship with the unfavorable outcome of AIS, which reduced the loss of information and quantified their relationship. Most covariates have complete information, with few missing. Second, compared with the previous research, the research on the nonlinearity addressing is a significant improvement. Third, Multiple imputation was employed to handle missing data. This method can maximize statistical power and minimize potential bias caused by covariate information missing. Besides, We performed a series of sensitivity analyses (target independent variable transformation, calculating E-values to explore the potential for unmeasured confounding, and reanalyzing the association between TG/HDL-c ratio and unfavorable outcomes of AIS after excluding participants with DM, TC ≥ 200 mg/dl, FBG ≥ 6.1 mmol/L, and BMI ≥ 25 kg/m^2^) to ensure the reliability of the results.

Potential limitations should be noted. First, the population included in this study is Korean. Therefore, the universality of these results for other races needs further verification. A second limitation is that the TG/HDL-c ratio was only measured at admission and not repeated thereafter. Therefore, we have no knowledge of whether the TG/HDL-c ratio changed 24 h after admission. It is an important question that may require further research. Third, some variable information was incomplete. For example, the original study database provided age-stratified information in ten intervals rather than patient-specific age, and this might result in incomplete variable information. We may consider designing our study in the future, and we will collect more detailed variable information. Fourth, as in all observational studies, even though known potential confounders factors were controlled, there might have been still uncontrolled or unmeasured confounders. Besides, this study is a secondary analysis based on published data, so variables that are not included in the data set cannot be adjusted, such as information on intravenous thrombolysis or endovascular thrombectomy. However, we calculated E-value to quantify the potential impact of unmeasured confounders and found that unmeasured confounders were unlikely to explain the results.

## Conclusion

This study demonstrated a nonlinear relationship and threshold effect between TG/HDL-c ratio and 3-month unfavorable outcomes in patients with AIS. When the TG/HDL-c ratio is lower than 3.515, the TG/HDL-c ratio is significantly negatively related to the risk of unfavorable outcomes. Their relationship was positively associated when the TG/HDL-c ratio is greater than 3.515. This provides a reference for optimizing lipidemia intervention and promoting clinical communication in patients with AIS.

## Supplementary Information

**Additional file 1:** **Table S1.** Collinearity screening. **Table S2.** Relationship between TG/HDL-c ratio and unfavorable outcome  3-month after stroke in participants with TG/HDL-c ratio≤3.515. **Table S3.** Effectsize of TG/HDL-c on unfavorable outcome in prespecified and exploratory subgroups. **Table S4.** Results of a two-piece linear regression model on the relationship between TG and HDL-cand unfavorable outcome.

## Data Availability

Data can be downloaded from the ‘PLos one’ database (https://journals.plos.org/plosone/).

## Abbreviations

TG/HDL-c: Triglyceride-to-high density lipoprotein cholesterol ratio
AIS: Acute ischemic stroke
HGB: Hemoglobin concentration
HCT: Hematocrit
MCV: Mean corpuscular volume
PLT: Platelet
TG: Triglyceride
TC: Total cholesterol
HDL-c: High-density lipoprotein cholesterol
LDL-c: Low-density lipoproteins cholesterol
BUN: Blood urea nitrogen
Scr: Serum creatinine
ALP: Alkaline phosphatase
ALT: Alanine aminotransferase
AST: Aspartate aminotransferase
ALB: Serum albumin
FBG: Fasting blood glucose
FIB: Fibrinogen
BMI: Body mass index
DM: Diabetes mellitus
CHD: Coronary heart disease
TIA: Transient ischemia attack
LAA: Large artery atherosclerosis
SVO: Small-vessel occlusion
CE: Cardio embolism
GAM: Generalized additive models
mRS: Modified Rankin Scale
OR: Odds ratio
CI: Confidence interval
