# Supplementary material for: Association between triglyceride-to-high density lipoprotein cholesterol ratio and three-month outcome in patients with acute ischemic stroke: a second analysis based on a prospective cohort study
Source: BMC Neurol. 2022 Jul 16;22:263. doi: 10.1186/s12883-022-02791-2 (PMC9287925; doi:10.1186/s12883-022-02791-2)
Supplement: Supplementary file 1 — Additional file 1: Table S1. Collinearity screening. Table S2. Relationship between TG/HDL-c ratio and unfavorable outcome 3-month after stroke in participants with TG/HDL-c ratio≤3.515. Table S3. Effectsize of TG/HDL-c on unfavorable outcome in prespecified and exploratory subgroups. Table S4. Results of a two-piece linear regression model on the relationship between TG and HDL-cand unfavorable outcome. [file 12883_2022_2791_MOESM1_ESM.docx]

**Association between triglyceride-to-high density lipoprotein cholesterol ratio and three-month** **outcome in Patients with Acute Ischemic Stroke: a second analysis based on a prospective cohort study**

**Running title:** **TG/HDL-c and Outcome**

Yong Han^1#^, Zhiqiang Huang^1^^#^,Jinsong Zhou ^2#^, Zhibin Wang^1#^ ,Qiming Li^1^, Haofei Hu^3*^, Dehong Liu^1*^

^1^ Department of emergency, Shenzhen Second People's Hospital, Shenzhen 518035, Guangdong Province, China

^2^ Department of laboratory medicine, Shenzhen Second People's Hospital, Shenzhen 518035, Guangdong Province, China

^3^ Department of nephrology, Shenzhen Second People's Hospital, Shenzhen 518035, Guangdong Province, China

Yong Han^1#^, Zhiqiang Huang^1#^, Jinsong Zhou ^2#^, and Zhibin Wang^1#^ have contributed equally to this work.

*Corresponding author

Haofei Hu^3#^

Department of nephrology, Shenzhen Second People's Hospital

No.3002 Sungang Road, Futian District,

Shenzhen 518035,

Guangdong Province,

China.

[huhaofei0319@126.com](mailto:huhaofei0319@126.com)

*Corresponding author

Dehong Liu

Department of emergency, Shenzhen Second People's Hospital

No.3002 Sungang Road, Futian District,

Shenzhen 518035,

Guangdong Province,

China

E-mail: dhliu_emergency@163.com

**Table S1 collinearity screening**

|  | Step 1 | Step 2 | Step 3 | Step 4 | Step 5 |
| --- | --- | --- | --- | --- | --- |
| TG/HDL ratio, | 12.5 | 12.4 | 12.4 | 2.1 | 1.8 |
| HGB(g/dl) | 667.8 | 168.8 | 168.8 | 168.8 | 168.8 |
| HCT(%) | 752.1 | 167.1 | 161.2 | 161.2 | 161.2 |
| TC(mg/dl) | 7.4 | 7.4 | 7.4 | 7.3 | NA |
| LDL-c(mg/dl) | 6.1 | 6.1 | 6.1 | 6.1 | NA |
| Scr(μmol/L) | 1.9 | 1.8 | 1.8 | 1.8 | 1.8 |
| TG (mg/dl) | 8.7 | 8.7 | 8.7 | NA | NA |
| HDL-c(mg/dl) | 3.9 | 3.9 | 3.9 | 2.7 | 1.9 |
| PLT(10^9/L) | 1.2 | 1.2 | 1.2 | 1.2 | 1.2 |
| MCV*(*fl*).* | 125.9 | 4.6 | 4.5 | 4.5 | 4.5 |
| BUN BUN(mg/dl) | 1.9 | 1.9 | 1.9 | 1.9 | 1.9 |
| AST(U/L) | 2 | 2 | 2 | 2 | 2 |
| ALT(U/L) | 1.9 | 1.9 | 1.9 | 1.9 | 1.9 |
| ALB ALB(g/dL) | 1.7 | 1.7 | 1.7 | 1.7 | 1.6 |
| FBG(mmol/L) | 1.4 | 1.4 | 1.4 | 1.4 | 1.4 |
| FIB（mg/L | 1.3 | 1.3 | 1.3 | 1.3 | 1.2 |
| BMI(kg/m2) | 1.2 | 1.2 | 1.2 | 1.2 | 1.2 |
| hypertension | 1.1 | 1.1 | 1.1 | 1.1 | 1.1 |
| DM | 1.4 | 1.4 | 1.4 | 1.4 | 1.4 |
| Previous stroke/TIA | 1 | 1 | 1 | 1 | 1 |
| Smoking | 1.1 | 1.1 | 1.1 | 1.1 | 1.1 |
| CHD | 1.1 | 1.1 | 1.1 | 1.1 | 1.1 |
| Stroke etiology | 1.1 | 1.1 | 1.1 | 1.1 | 1.1 |
| NIHSS score | 1.2 | 1.2 | 1.2 | 1.2 | 1.2 |

**NA was the excluded variable**

HGB, hemoglobin concentration; HCT, Hematocrit; MCV, mean corpuscular volume; PLT, platelet*;* TG, triglyceride; TC, total cholesterol; HDL-c, high-density lipoprotein cholesterol; LDL-c, low-density lipoproteins cholesterol; BUN, blood urea nitrogen; Scr, serum creatinine*;* ALT, alanine aminotransferase; AST, aspartate aminotransferase; ALB, serum albumin*;* FBG, fasting blood glucose; FIB, fibrinogen*;* BMI *,* body mass Index; DM, diabetes mellitus*;* CHD, coronary Heart Disease; TIA, transient ischemia attack . NIHSS, national Institute of health stroke scale; TG/HDL ratio, triglyceride -to- high density lipoprotein ratio.

**Table S2. Relationship between TG/HDL-c ratio and unfavorable outcome 30 days after stroke in participants with TG/HDL-c ratio≤3.515**

| Exposure | Crude model (OR,95%CI) P | Model I(OR,95%CI) P | Model II(OR,95%CI) P |
| --- | --- | --- | --- |
| TG/HDL-c ratio | 0.779 (0.666, 0.912) 0.002 | 0.836 (0.711, 0.984) 0.031 | 0.774 (0.634, 0.947) 0.002 |

Crude mode1: we did not adjust other covariates

Model I: we adjusted age, sex

Model II: we adjusted age, sex, HGB, BMI, HCT, AST, BUN, ALB, FBG, FIB, DM, previous stroke or TIA, hypertension, CHD, stroke etiology, smoking, NIHSS score.

**Table S3. Effect size of TG/HDL-c on unfavorable outcome in prespecified and exploratory subgroups**

| Characteristic | No of participants | OR (95%CI) P value P for interacion |
| --- | --- | --- |
| Age（years） |  | 0.9768 |
| <60 | 395 | 0.977 (0.789, 1.209) 0.830 |
| 60 to <70 | 466 | 0.926 (0.763, 1.124) 0.438 |
| 70 to <80 | 629 | 0.934 (0.801, 1.088) 0.379 |
| ≥80 | 274 | 0.966 (0.795, 1.172) 0.723 |
| Gender |  | 0.7477 |
| Male | 1073 | 0.959 (0.855, 1.076) 0.475 |
| Female | 691 | 0.988 (0.856, 1.140) 0.868 |
| TC（mg/dL） |  | 0.2084 |
| <200 |  | 0.989 (0.887, 1.101) 0.8340 |
| ≥200 |  | 0.876 (0.747, 1.026) 0.1015 |
| BMI (kg/m^2^) |  | 0.4349 |
| <18.5 | 95 | 0.539 (0.235, 1.235) 0.144 |
| ≥18.5, < 25 | 1144 | 0.933 (0.835, 1.042) 0.219 |
| ≥25 | 525 | 0.914 (0.772, 1.082) 0.298 |
| FBG (mmol/L) |  | 0.4349 |
| <6.1 | 1264 | 0.933 (0.830, 1.048) 0.240 |
| ≥6.1 | 560 | 0.972 (0.845, 1.118) 0.692 |
| DM |  | 0.2511 |
| No | 1213 | 0.903 (0.801, 1.018) 0.096 |
| Yes | 551 | 1.002 (0.877, 1.146) 0.971 |

Note 1:Above model adjusted for age, sex, HGB, BMI, HCT, AST, BUN, ALB, FBG, FIB, DM, previous stroke or TIA, hypertension, CHD, stroke etiology, smoking, NIHSS score.

Note 2:In each case, the model is not adjusted for the stratification variable

OR, odds ratios; CI: confidence interval;

**TableS4. Results of a two-piece linear regression model on the relationship between TG and HDL-c and unfavorable outcome**

| unfavorable outcome: | Model I (OR,95%CI) P |  | Model II(OR,95%CI) P |
| --- | --- | --- | --- |
| Fitting model by standard linear regression | 0.998 (0.995, 1.001) 0.1775 |  | 1.006 (0.996, 1.016) 0.2517 |
| Fitting model by two-piecewise linear regression | |  |  |
| Inflection point of TG or HDL-c(mg/dl) | 95 |  | 45 |
| ≤95 | 0.990 (0.981, 0.999) 0.025 0.002 | ≤45 | 1.011 (0.996, 1.026) 0.1358 |
| > 95 | 1.000 (0.997, 1.004) 0.806 | >45 | 1.017 (0.983, 1.052) 0.3364 |
| P for log-likelihood ratio test | 0.058 |  | 0.337 |

OR, odds ratios; CI: confidence, Ref: reference;

**M**odel **I** :TG and **unfavorable outcome,** we adjusted age, sex, HGB, BMI, HCT, AST, BUN, ALB, FBG, FIB, DM, previous stroke or TIA, hypertension, CHD, stroke etiology, smoking, and NIHSS score.

**M**odel **II** :HDL-c and **unfavorable outcome,** we adjusted age, sex, HGB, BMI, HCT, AST, BUN, ALB, FBG, FIB, DM, previous stroke or TIA, hypertension, CHD, stroke etiology, smoking, and NIHSS score.
